# Retraction Note: Protocatechuic aldehyde from Salvia miltiorrhiza exhibits an anti-inflammatory effect through inhibiting MAPK signalling pathway

**DOI:** 10.1186/s12906-023-03984-z

**Published:** 2023-05-05

**Authors:** Shuang Wu, Qingyu Wang, Jinquan Wang, Baoyu Duan, Qihe Tang, Zhuojian Sun, Jinlong Han, Chenggang Shan, Zhifen Wang, Zhihui Hao

**Affiliations:** 1 Agricultural Bio-Pharmaceutical Laboratory, Qingdao Agricultural University, Qingdao, 266109 China; 2 National Centre for Veterinary Drug Safety Evaluation, College of Veterinary Medicine, Agricultural University, Beijing, 100089 China; 3 College of Animal Medicine, Xinjiang Agricultural University, Wulumuqi, 830001 China; 4 College of Medical Technology, Shanghai University of Medical and Health Sciences, Shanghai, 201318 China; 5 Agricultural Products Processing Institute of Shandong Academy of Agricultural Sciences, Jinan, 250000 China; 6 Center of Research and Innovation of Chinese Traditional Veterinary Medicine, College of Veterinary Medicine, China Agricultural University, Beijing, 100089 China

**Retraction Note: BMC Complement Med Ther 20, 347 (2020)**

**https://doi.org/10.1186/s12906-020-03090-4**

The Editor has retracted this article. After publication, concerns were raised regarding the western blot images. Specifically:

In Fig. 6, the blots representing p38 and p-p38 appear highly similar.

In the Supplementary materials, the raw data provided for the blots presented in Figs. 5 and 6 appear to contain straight-line breaks and repetitive patterns in the backgrounds.

Additionally, there appear to be irregularities in the chromatogram traces presented in Fig. 1.

The authors have provided a replacement blot for p38 and stated that the image irregularities were caused by technical problems. However, further raw data provided by the authors appear different from the published versions of the figures and Supplementary materials. The Editor therefore no longer has confidence in the presented data.

Shuang Wu has stated on behalf of all co-authors that they agree to this retraction.

